# The crystal structure of superoxide dismutase from *Plasmodium falciparum*

**DOI:** 10.1186/1472-6807-6-20

**Published:** 2006-10-04

**Authors:** Ian W Boucher, Andrzej M Brzozowski, James A Brannigan, Claudia Schnick, Derek J Smith, Sue A Kyes, Anthony J Wilkinson

**Affiliations:** 1 Structural Biology Laboratory, Department of Chemistry, University of York, York YO10 5YW, UK; 2 Molecular Parasitology Group, Weatherall Institute of Molecular Medicine, John Radcliffe Hospital, Headington, Oxford OX3 9DS, UK; 3 Bioinformatics Institute, 30 Biopolis St., Singapore 138671, Singapore

## Abstract

**Background:**

Superoxide dismutases (SODs) are important enzymes in defence against oxidative stress. In *Plasmodium falciparum*, they may be expected to have special significance since part of the parasite life cycle is spent in red blood cells where the formation of reactive oxygen species is likely to be promoted by the products of haemoglobin breakdown. Thus, inhibitors of *P. falciparum *SODs have potential as anti-malarial compounds. As a step towards their development we have determined the crystal structure of the parasite's cytosolic iron superoxide dismutase.

**Results:**

The cytosolic iron superoxide dismutase from *P. falciparum *(*Pf*FeSOD) has been overexpressed in *E. coli *in a catalytically active form. Its crystal structure has been solved by molecular replacement and refined against data extending to 2.5 Å resolution. The structure reveals a two-domain organisation and an iron centre in which the metal is coordinated by three histidines, an aspartate and a solvent molecule. Consistent with ultracentrifugation analysis the enzyme is a dimer in which a hydrogen bonding lattice links the two active centres.

**Conclusion:**

The tertiary structure of *Pf*FeSOD is very similar to those of a number of other iron-and manganese-dependent superoxide dismutases, moreover the active site residues are conserved suggesting a common mechanism of action. Comparison of the dimer interfaces of *Pf*FeSOD with the human manganese-dependent superoxide dismutase reveals a number of differences, which may underpin the design of parasite-selective superoxide dismutase inhibitors.

## Background

Superoxide dismutases (SODs) are crucial enzymes in both eukaryotes and prokaryotes. They catalyse the dismutation of the superoxide radical to hydrogen peroxide and dioxygen according to the two-step reaction as follows:

M^3+ ^+ O_2 _^.- ^→ M^2+ ^+ O_2_

M^2+ ^+ O_2 _^.- ^+ 2H^+ ^→ M^3+ ^+ H_2_O_2_

where M denotes a metal ion which interconverts between oxidised and reduced states. The superoxide radical O_2 _^.- ^is formed in cells as a result of both enzymatic and spontaneous oxidation reactions. The superoxide radical is toxic to living cells as it oxidises and degrades biological molecules such as lipids and proteins [1].

For many years it was thought that malaria parasites had no requirement for an endogenous superoxide dismutase and merely exploited the activity of the host's enzyme in the red blood cell [2]. However, in 1996 a *Plasmodium falciparum *iron-dependent SOD (*Pf*FeSOD) was identified in parasites isolated from infected blood cells [3]. Malaria parasites are particularly prone to oxidative damage in the intra-erythrocytic stage of their lifecycle. This is because an important source of amino acids for the parasite is red blood cell haemoglobin. Haemoglobin degradation produces free haem groups leading to oxidation of the iron from the ferrous (Fe^2+^) to the ferric (Fe^3+^) state. This oxidation liberates electrons, which promote the formation of reactive oxygen intermediates, including superoxide. The *Pf*FeSOD gene is expressed at its highest level during this stage of the parasite life cycle [4].

SODs are classified according to their metal cofactors. Eukaryotic cells are generally served by a cytosolic Cu/ZnSOD and an evolutionarily unrelated mitochondrial MnSOD. Some eukaryotes also contain a SOD containing a single Fe atom. Bacterial cells feature single metal-centred SODs in which the metal can be either manganese or iron. The FeSODs and the MnSODs exhibit recognisable similarities in their sequences, they have a common α/β tertiary structure and they use the same residues to coordinate the metal [5]. These are quite distinct from the two-metal Cu/ZnSODs, which have a Greek key β-barrel fold [6]. The MnSODs of eukaryotic origin are distinguishable from those of prokaryotic sources on the basis of their quaternary structure; the former are tetramers while the latter are dimers. Small sequence differences distinguish the Mn- and FeSODs [7]. Biochemically, FeSODs are more sensitive to inhibition by azide [8] and have a greater susceptibility to inactivation by hydrogen peroxide, than MnSODs. Iron and manganese superoxide dismutases can bind either metal cofactor. However, most are only functional with their cognate metal co-factor bound. Some enzymes however, maintain activity with either Fe or Mn bound and are termed cambialistic [9].

In 2002, an electron paramagnetic resonance and modelling study of SOD from *P. falciparum *showed it was, as expected, an iron-dependent dimer [10]. The fact that it is an FeSOD and distinct from human tetrameric Mn and Cu/ZnSODs raises the possibility of its exploitation as an anti-malarial drug target [11] and indeed inhibitors of *Pf*FeSOD have been identified [12]. This study presents an X-ray crystal structure of *P. falciparum *FeSOD solved at a resolution of 2.5 Å.

## Results

### Protein characterization

Purified *Pf*FeSOD was resolved by electrophoresis for 3 hours at 100 V in a 10% native polyacrylamide gel. The gel was soaked in riboflavin and NADPH to generate superoxide radicals, and stained by Nitro Blue Tetrazolium (NBT) dye. NBT reacts with superoxide to form a blue precipitate when fixed in a gel. Any regions on the gel that exhibit SOD activity will therefore be white against a blue background. A clear white band coincident with the *Pf*FeSOD protein indicated that the protein was active. Analytical ultracentrifugation (AUC) sedimentation equilibrium experiments estimated the molecular weight of the protein to be 47,400 ± 700 Da. Since the calculated molecular weight of the monomer is 23,799 Da, this indicates that *Pf*FeSOD is a dimer, consistent with previously characterised FeSODs, which are either dimers or tetramers.

### Overall structure

The crystal structure of *Pf*FeSOD has been solved and refined against data extending to 2.5 Å spacing. The refined model consists of two polypeptide chains, A and B, which are related by a non-crystallographic two-fold axis of symmetry, together with two iron ions and 140 solvent water molecules. The electron density maps are contiguous from residue 1 to residue 197 of both chains. The *C*-terminal *Pf*FeSOD residue (Lys^198^), together with the eight residues of the *C*-terminal tag are not observed in the electron density maps. Details of the refinement statistics are given in Table 1. There is a solitary outlier in the Ramachandran plot at residue Lys^43 ^in the B chain, which occurs in a partially ordered surface loop. The Ser^15^-Pro^16 ^peptide bond in both chains is in the *cis *conformation.

**Table 1:** Data collection and refinement statistics

	**PfFeSod (PDB code 2BPI)**
*Data collection*	
X-ray source	ESRF Beamline ID29
Wavelength (Å)	0.976000
Collection Temperature (K)	100
Resolution range (Å)	25.00 – 2.52
Space group	*P*2_1_2_1_2_1_
Unit-cell parameters (Å)	*a *= 55.94, *b *= 78.91, *c *= 90.62
Matthews coefficient/solvent content (%)	2.2/43.0
Number of unique reflections, overall/outer shell^a^	13247 (843)
Completeness (%), overall/outer shell^a^	98.8 (100)
Redundancy, overall/outer shell^a^	4.1 (4.2)
*I*/σ(*I*), overall/outer shell^a^	17.3 (6.2)
*R*_merge_^b ^(%), overall/outer shell^a^	8.3 (29.7)
	
*Refinement and model statistics*	

*R*-factor^c^	0.187 (0.224)
*R*-free^d^	0.263 (0.338)
Molecules/asymmetric unit	2
Number of protein non hydrogen atoms	3345
Number of water molecules	140
Rms deviation from target^e^	
Bond lengths (Å)	0.014
Bond angles (°)	1.453
Average *B*-factor (Å^2^)	28.10
Ramachandran plot^f^	90.3/8.6/0.6/0.6

^a ^Figures in parentheses concern the outer shell and corresponds to 2.582–2.520 Å.

^b ^*R*_merge _= Σ_*hkl *_Σ_*i*_|*I*_*i *_- <*I*> |/Σ_*hkl *_Σ_*i *_<*I*> where *I*_*i *_is the intensity of the *i*th measurement of a reflection with indexes *hkl *and *<I> *is the statistically weighted average reflection intensity.

^c ^*R*-factor = Σ ||*F*_*o*_| - |*F*_*c*_||/Σ |*F*_*o*_| where *F*_*o *_and *F*_*c *_are the observed and calculated structure factor amplitudes, respectively.

^d ^*R*-free is the *R*-factor calculated with 5 % of the reflections chosen at random and omitted from refinement.

^e ^Root-mean-square deviation of bond lengths or bond angles from ideal geometry.

^f ^Percentage of residues in most favoured/additionally allowed/generously allowed/disallowed regions of the Ramachandran plot, according to PROCHECK.

The overall structure is similar to those of previously solved iron superoxide dismutases, consisting of an amino terminal α-helical domain linked to a carboxy terminal α/β domain. The first eleven residues have an extended structure and are involved in crystal packing. The next 70 residues form two long anti-parallel α-helices (α1 and α3) connected by a meandering loop containing a third helix, α2. In both subunits, helix α1 has a noticeable kink at the conserved Lys^29 ^residue, a feature that has been noted before [13]. This domain is connected via an extended loop to the α/β domain (Figure 2A), which comprises a three-stranded anti-parallel β-pleated sheet (strand order β3-β1-β2) surrounded by four α-helices. Superposition of equivalent Cα atoms of chain A and chain B of *Pf*FeSOD gives a positional root mean squared deviation (rmsΔ) of 0.3 Å. The *Pf*FeSOD structure superposes onto the molecular replacement model, *E. coli *FeSOD, with an rmsΔ of 0.55 Å over all equivalent Cα atoms.

**Figure 1:**
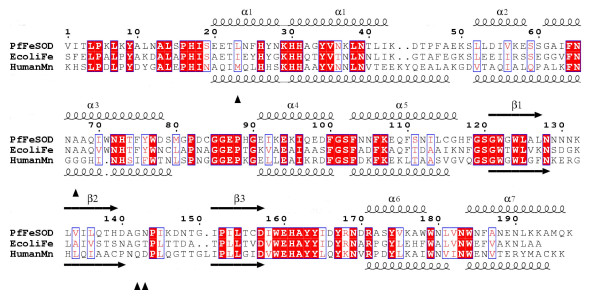
Sequence alignment of FeSOD from *P. falciparum *and *E.coli *and MnSOD from the human mitochondrion. Identical residues are highlighted with a red background and similar residues are boxed. *Pf*FeSOD (top) and MnSOD (PDB code 1n0j [36] bottom) secondary structure elements are indicated. Residues predicted to be specific to MnSOD sequences [37] are marked with a triangle. Note the similarity of secondary structure between the two types of SOD. The longer helices in the *N*-terminal domain of MnSOD contribute to tetramer formation. The figure was made using ESPript [38].

**Figure 2:**
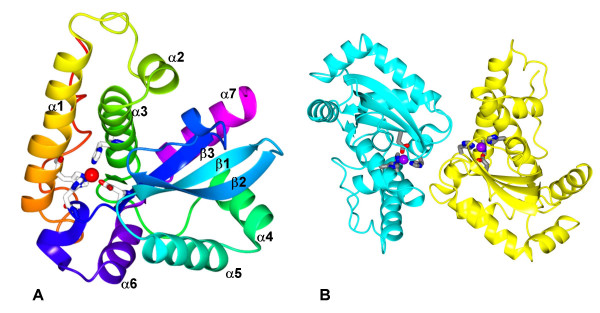
**A) **Ribbon representation of *Pf*FeSOD polypeptide chain with colour ramping from the amino terminus in red to the carboxyl terminus in magenta. The iron is shown as a red sphere and its coordinating residues His^26^, His^73^, Asp^157 ^and His^161 ^are shown in cylinder representation. **B) **The *Pf*FeSOD dimer coloured by subunit with the Fe atoms as mauve spheres and their coordinating residues in cylinder representation. The figures were made using CCP4 mg [39].

### Structural comparisons and dimer interface

The tertiary structure is very similar to that of a number of other Fe- or Mn-dependent SOD enzymes. Of particular interest is comparison with the tetrameric MnSOD found in human mitochondria. Subtle differences in the structures may allow the design of inhibitors to block the activity of FeSOD selectively without affecting the MnSOD. The arrangement of the active site residues is well conserved between the Fe and MnSODs; the only significant difference in main chain conformation arises in the loop connecting α1 and α3, which is remote from the active site and the dimer interface.

As is typical of SOD proteins, only a relatively small accessible surface area (9.7 %) becomes buried in the dimer interface. The main contributions to the dimer interface arise from helix α1, the loop that connects α5 to β1 and the loop connecting β3 to α6. 41% of the atoms forming the dimer interface are polar, while 59% are non-polar. There are five bridging water molecules within the dimer interface. There is an interesting pair of adjacent charge-charge interactions between the carboxylates of the Asp^140 ^residues and the side chain imidazoles of His^139^. Perhaps associated with this, the dihedral angles of residue Asp^140 ^appear in a generously allowed location on the Ramachandran plot. These residues lie on what would be a classical β-turn were it not for the fact that residue 141 is an alanine rather than a glycine. All of the dimer stabilising interactions in the *E. coli *enzyme are conserved in *Pf*FeSOD. Such high conservation of the dimer interface reflects the importance of dimerisation to the activity of dismutases.

### Active site of *P. falciparum *FeSOD

The metal ion in both subunits of the enzyme is five-coordinate. Three bonds arise from coordination with histidine Nε2 atoms of residues His^26 ^(mean Fe-Nε2 distance 2.2 Å), His^73 ^(2.1 Å) and His^161 ^(2.0 Å). A further bond is between the metal and the Oδ2 atom of Asp^157 ^(mean Fe-Oδ2 distance 1.9 Å). The fifth coordination position is an oxygen atom from either a water molecule or a hydroxide species (mean Fe-O distance 2.1 Å). The coordination geometry is trigonal bipyramidal, with His^73^, His^161 ^and Asp^157 ^providing the equatorial ligands and with His^26 ^and the hydroxide or water oxygen atom forming the axial ligands.

The area around the active site is fairly hydrophobic with two tryptophan residues (122 and 159) 5.4 Å and 6.1 Å from the metal, respectively. Trp^122 ^forms a hydrogen bond to Gln^69^, which in turn forms a hydrogen bond with the hydroxyl group of Tyr^34^. Tyr^34 ^is also hydrogen bonded via a water molecule to His^30^, which is bonded to Tyr^134 ^from the other subunit. This classical hydrogen-bonding network in superoxide dismutases may allow communication between the two metal sites [13]. In the B chain however, we note that the Gln^69 ^Nδ to Tyr^34 ^-OH distance is longer (3.6 Å), perhaps due to coordinate error at this resolution. The spatial arrangement of the residues in the active site is otherwise identical to those of other Fe-dependent superoxide dismutases whose structures are known, consistent with the notion that structure and mechanism are highly conserved in this enzyme class.

A second line of communication between the active sites is afforded by the intermolecular ionic/hydrogen bonding interaction between the Fe-coordinating His^161 ^and the carboxylate of Glu^160^. The latter is situated in a loop that links two Fe-coordinating residues (Asp^157 ^and His^161^) in the opposing subunit. The relevance, if any, of structural communication between the active sites to the kinetics and mechanism of these enzymes is not established.

### Modelling of the *P. falciparum *mitochondrially targeted FeSOD

The sequence of the major FeSOD is highly conserved in different Plasmodia species (see Additional file 2) and is encoded by a gene on chromosome 8 (PlasmoDB code PF08_0071 [14]) consisting of a single exon. Another gene product (*Pf*FeSOD2) with significant sequence homology is located on chromosome 6. This *Pf*FeSOD2 gene is interrupted with multiple introns. The original gene model (MAL6P1.194 [14]) was revised by a bioinformatic analysis to maximise primary sequence homology to *Pf*FeSOD, leading to a gene predicted to consist of nine exons, separated by eight introns with consensus splice donor/acceptor sites (see Additional file 3). Evidence supporting the model was found by *Plasmodium *interspecies conservation of the intron-exon structure, which is a good predictor for small exons [15] and subsequently confirmed by cDNA cloning [16]. A striking feature of the *Pf*FeSOD2 gene is that it encodes an *N*-terminal sequence typical of proteins that are targeted to sub-cellular organelles. The first two exons coincide precisely with elements of the bipartite signal (see Additional file 4) namely a signal sequence and a transit peptide. A GFP-fusion with the signal is directed to the mitochondrion, despite predictions from the sequence that it would be delivered to the apicoplast [16]. However, the gene structure not only hints at its evolution by exon shuffling, but provides the opportunity for alternatively spliced forms [17].

A homology model (see Additional file 1) of the mitochondrially targeted *Pf*FeSOD2 was derived using the cytosolic *Pf*FeSOD structure solved here. The cytosolic *Pf*FeSOD is the closest in sequence of any dismutase to the mitochondrial *Pf*FeSOD and hence provides the most suitable coordinate set from which to construct a homology model. The model generated has an rmsΔ on all Cα atoms of 0.36 Å for the 388 matched residues. Unsurprisingly the secondary structure is predicted to be identical to that of the cytosolic FeSOD of *Plasmodium*. It appears that the active site and dimer interface are also well conserved. This is consistent across the Fe/MnSOD enzyme family, which is a highly conserved enzyme class. It remains to be seen if the two superoxide dismutases from *Plasmodium *are indeed structurally and biochemically equivalent, or whether the mitochondrially targeted SOD has a different turnover/stability that is required for function within a specific environment in the cell.

## Discussion

Superoxide dismutases play a crucial role in the chemical protection strategies many cells adopt to counter the considerable threat posed by the superoxide anion radical. Iron-containing superoxide dismutases are essentially confined to prokaryotes, algae, higher plants and certain protozoa [12]. In contrast, the human superoxide dismutases are either Mn- or Cu/Zn-dependent. Thus, selective inhibitors of FeSODs have potential uses in therapy against diseases caused by pathogens. At first sight it would appear difficult to inhibit *Pf*FeSOD using classical 'active-site directed' approaches. The substrate-binding site is small, it needs to accommodate just two atoms and the design of selective inhibitors may be hampered by the structural similarity that the iron-containing superoxide dismutases share with the manganese-dependent enzymes. Superoxide is attracted to the active site of dismutases through a funnel, which comprises residues from both subunits in the dimer. The funnel lies at the top of the dimer interface near to the metal sites. It is thought that potential inhibitors could bind here and block access to the active site for the superoxide substrate [13].

An alternative approach is to target inhibitors towards the subunit interface so as to disrupt the quaternary structure. A possible explanation of the widely accepted "ping-pong" kinetics observed for FeSODs would be if superoxide binding in one active site is accompanied by oxidation to oxygen in the other, with the iron atoms in the respective subunits maintaining opposing oxidation states as they alternate between the +2 and +3 forms. If so, dimerisation would likely be important to the mechanism of FeSOD, and monomeric FeSODs are not found in Nature [13]. However, evidence from Cu/ZnSODs suggests that monomeric forms are active which may mean that dimerisation is not crucial for the function of all superoxide dismutases [18]. Despite this, it is clear that the dimer interface in Fe and MnSODs is important in the SOD reaction with dimer interface mutants of human MnSOD exhibiting reduced steady-state catalytic constants [19,20]. Other evidence points to the importance of dimerisation for functional SOD activity, as dimer destabilising mutations of Cu/Zn SOD have been linked to motor neuron disease [21]. Although the mechanism is unclear, the destabilised mutants may be more prone to aggregation, which is commonly associated with the disease.

There are a number of significant differences between the dimer interfaces in *Pf*FeSOD and HuMnSOD that may be exploited in the design of parasite selective inhibitors. Residue Phe^118^, which contributes 15 % of the accessible surface area buried in the interface in *Pf*FeSOD is replaced with glutamine in the human enzyme. The intermolecular polar/salt-bridging residue pair of His^139 ^and Asp^140 ^in *Pf*FeSOD is substituted by Cys and Pro, respectively in HuMnSOD, similarly the Glu^21^-Arg^168 ^ion pair in *Pf*FeSOD appears as Ala and Lys respectively in the human enzyme. These differences ought to be exploitable in the selective design of dimer-disrupting mutants.

Inhibitors of *Pf*FeSOD have already been identified from a random screening of a chemical library of compounds [12]. A subset of these uncharged polyaromatic compounds, with molecular weights in the range 200–700 Da, have *in vitro *anti-malarial activity, with IC_50 _values in the micromolar range. Their mechanism of action and their selectivity was not determined so it remains to be seen whether they interfere with dimer formation. A number of small molecule inhibitors of protein-protein interactions with biomedical significance have been reported recently [22]. For *Trypanosoma cruzi*, a parasite-selective inhibitor of triosephosphate isomerase has been identified and shown to act by blocking dimerisation [23], so it appears that the strategy could be useful, particularly as the percentage of accessible protein surface that becomes buried in a SOD dimer is at the lower limit of the range normally seen in protein multimers in Nature.

## Conclusion

The X-ray structure of *P. falciparum *SOD provides the detailed template on which comparisons with human enzyme can be made, and for the design of potential parasite-specific inhibitors. In particular, the biochemical properties of the SOD enzyme (and differences to the human SOD protein) suggest that a possible mode of inhibition would be to disrupt the dimer interface that may be important to its function and stability. The intra-erythocytic stages of the parasite are in an environment that is known to be rich in antioxidant defences (both chemical and enzymatic) that might compensate an inhibitory effect. However, the selective delivery of inhibitors to the parasite organellar system may prove to be a useful route to new therapeutics.

## Methods

### Cloning and expression of *P. falciparum *FeSOD

The cytosolic FeSOD coding sequence was amplified from *P. falciparum *(3D7) genomic DNA using Vent™ polymerase (New England Biolabs) and the oligonucleotide primers:

5'-CATG*CCATGG*TTATTACATTGCCCAAATTAAAG-3'

5'-CCG*CTCGAG*CTTTTGCATAGCTTTTTTTAAGTT-3'.

The 597 base pair PCR product was digested with *Nco*I and *Xho*I and ligated to similarly cut pET28a (Novagen). This places the coding sequence for *Pf*FeSOD upstream of that for a non-cleavable, *C*-terminal His_6 _tag. The expected expression product therefore comprises residues 1–198 of *Pf*FeSOD with a *C*-terminal LEHHHHHH tag. The ligation products were introduced into competent *E. coli *NovaBlue™ cells (Novagen). Kanamycin-resistant transformants harbouring pET28-*Pf*FeSOD were identified and isolated. Plasmid DNA from these cells was purified and subsequently introduced into the expression strain *E. coli *BL21-CodonPlus (Stratagene). An overnight culture of *E. coli *BL21/pET28-*Pf*FeSOD was used to inoculate 1 litre of Luria-Bertani medium supplemented with 30μg ml^-1 ^kanamycin and cells were grown with shaking at 37°C to an OD_600 _of 0.6 at which point expression of recombinant protein was induced by the addition of isopropyl-β, D-thiogalactopyranoside to a final concentration of 1 mM. After a further 4 hours incubation at 30°C, cells were harvested by centrifugation.

### Purification, characterisation and crystallisation

The harvested cell pellet was resuspended in 50 mM Tris-HCl buffer pH 7.5 containing 300 mM NaCl (Buffer A). The cells were lysed by sonication and clarified by centrifugation. The supernatant was mixed with 2 ml Ni-NTA agarose resin (Qiagen) at 4°C for 1 hour with gentle shaking. The resin was then washed with Buffer A containing 20 mM imidazole and protein was eluted with Buffer A containing 100 mM imidazole. Fractions were examined by Coomassie staining following SDS-polyacrylamide gel electrophoresis and those containing proteins of approximately 23 kDa corresponding to FeSOD were pooled and further fractionated by size exclusion chromatography on a Superdex 75 (Pharmacia) column run in 50 mM Tris-HCl pH 7.5 containing 100 mM NaCl. Typically 10 – 15 mg of protein was obtained per litre of cell culture.

Sedimentation equilibrium experiments were conducted on a Beckman Optima XL/I analytical ultracentrifuge, in an AN-50Ti rotor. The buffer density (300 mM NaCl, 50 mM Tris-HCl, pH 7.5) and the partial specific volume of the protein were estimated to be 1.012 g.ml^-1 ^and 0.7302 respectively, using the program SEDNTERP [24]. Protein homogeneity was examined on an 8.75 % native polyacrylamide gel. Enzyme activity was confirmed using a gel-based Nitro blue tetrazolium assay [25].

Crystallisation experiments were carried out by the hanging-drop vapour-diffusion method. Drops contained a 1:1 volume ratio of *Pf*SOD at 10 mg ml^-1 ^in 50 mM Tris-HCl pH 7.5, 100 mM NaCl and a range of precipitants. Rod shaped crystals appeared within a week at 18°C from reservoir solutions that contained 0.1 M Tris-HCl pH 7.5 and 30–40% (*w/v*) polyethylene glycol (PEG) 600.

### Data collection

Crystals were mounted in a fine rayon loop and transferred into a solution containing 0.1 M Tris-HCl (pH 7.5) and 40 % PEG 600 which acts as a cryoprotectant. They were then flash-cooled in liquid nitrogen. Preliminary in-house X-ray analysis of these crystals showed that they belong to the space group *P*2_1_2_1_2_1 _with unit cell dimensions *a *= 55.5 Å, *b *= 78.4 Å, *c *= 89.3 Å. A complete X-ray diffraction data set was collected at 100 K, using synchrotron radiation at a wavelength of 0.976 Å, on beamline ID-29 at the ESRF, Grenoble, using an ADSC Quantum4 CCD detector. The data were processed using the programs *DENZO *and *SCALEPACK *[26].

### Structure determination and refinement

Calculations were performed using the *CCP4 *suite of programs [27]. Molecular replacement was performed with *AMoRe *[28] and refinement conducted with *REFMAC *[29]. The structure was determined by the molecular replacement method using the *Escherichia coli *FeSOD coordinate set (PDB entry code 1ISC) as a search model. *Ec*SOD and *Pf*SOD share 51% sequence identity. Molecular replacement calculations produced two clear solutions related by a non-crystallographic 2-fold symmetry axis, and giving an R-factor of 40.4 % after rigid body refinement. Model building was performed using the *X-AUTOFIT *routines [30] implemented in the molecular graphics programme *QUANTA *(Accelrys, San Diego). During refinement, tight non-crystallographic symmetry restraints were imposed on the main chain atoms and medium restraints on the side chains of the two molecules in the dimer.

### Modelling the targeted SOD

A homology model of the mitochondrial FeSOD (*Pf*FeSOD2) was generated on the basis of 52% sequence identity over 188 residues to the known structure of the cytosolic *Pf*FeSOD. All structural visualisation/manipulation was performed using QUANTA. The sequences of *Pf*FeSOD2 and the *Pf*FeSOD template structure were aligned using CLUSTALX [31], and manually corrected for certain regions, based on interrogation of the known structure.

Homology modelling was performed using MODELLER6 [32]. Ten initial models were generated, and the model with the lowest objective function chosen as the representative *Pf*FeSOD2 structure. After manual checking of backbone and side chain positions, energy minimization was performed using CHARMM [33,34] to relax steric clashes within the model. Stereochemical evaluation was then performed using PROCHECK [35]. The model possesses good stereochemical quality, with 88.4 % of residues in the most favoured regions of the Ramachandran map, and 10.4 % in additional allowed regions. There are four residues in generously allowed or unfavourable regions, which accurately reflect those in the template structure.

## Authors' contributions

JAB and IB conceived the study and together with AJW, who coordinated the project, drafted the manuscript; SK provided genomic DNA and advice on cloning; IB expressed the protein with JAB and purified the protein together with CS; IB carried out the crystallographic analysis under the guidance of AMB; DJS carried out the homology modelling work. All authors have approved the final manuscript.

**Figure 3:**
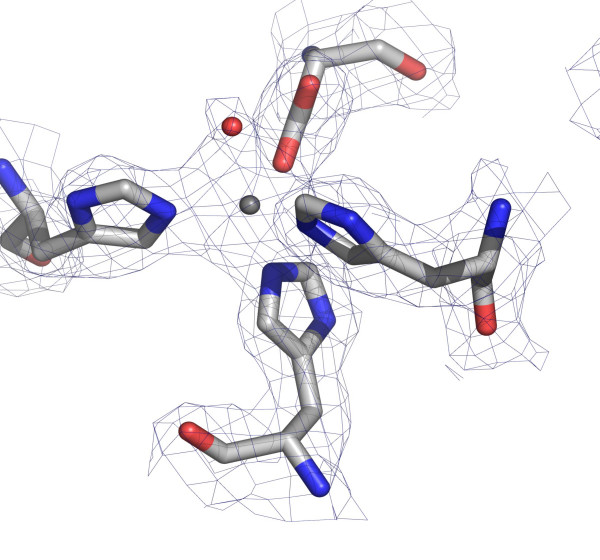
The active site from one subunit of the FeSOD dimer. The metal is shown as a grey sphere and the water molecule that acts as an axial ligand to the metal is coloured red. The *2F*_*o*_-*F*_*c *_electron density map, contoured at the one sigma level, is represented as a mesh.

## Supplementary Material

Additional File 1 Model of the *P. falciparum *mitochondrially targeted FeSOD2. Data file PfSOD2_model.pdb. Click here for file

Additional File 2 Sequence alignment of Plasmodia FeSOD sequences. FeSOD is one the most highly conserved proteins across different Plasmodium species [40]. Identical residues are highlighted with a red background and similar residues are boxed. *Pf*FeSOD secondary structure elements are superposed. Codes: Pf, *P. falciparum*; Pr, *P. reichenowi*; Po, *P. ovalae*; Pm, *P. malariae*; Pv, *P. vivax*; Pk, *P. knowlesi*; Py, *P. yeolii*; Pb, *P. berghei*; Pc, *P. chabaudi*; Pg, *P. gallinaceum*. The figure was made using ESPript [38]. Click here for file

Additional File 3 Gene model of *Pf*FeSOD2. A region of DNA sequence from *P. falciparum *chromosome 6 is shown, with predicted introns highlighted in yellow, and the protein product of the exon sequences in red. Click here for file

Additional File 4 Sequence alignment of predicted *Pf*FeSOD2 proteins from Plasmodium species. Identical residues are highlighted with a red background and similar residues are boxed. *Pf*FeSOD2 predicted secondary structure elements are superposed and exon boundaries are marked with a triangle. Note that the first two exons coincide with the putative signal sequence and transit peptide. Sequence data were gleaned from PlasmoDB [14]. Although not shown, the incomplete sequence data from *P. chabaudi *(missing region around exons 4 & 5) and *P. gallinaceum *(missing region encompassing 6 & 7) also show the same conserved pattern of putative intron boundaries. Codes: Pf, *P. falciparum*; Pr, *P. reichenowi*; Pv, *P. vivax*; Pk, *P. knowlesi*; Pb, *P. berghei*; Py, *P. yeolii*. Click here for file
