# Supplementary figures and images for: The crystal structure of superoxide dismutase from Plasmodium falciparum
Source: BMC Struct Biol. 2006 Oct 4;6:20. doi: 10.1186/1472-6807-6-20 (PMC1618392; doi:10.1186/1472-6807-6-20)

## Slide 1
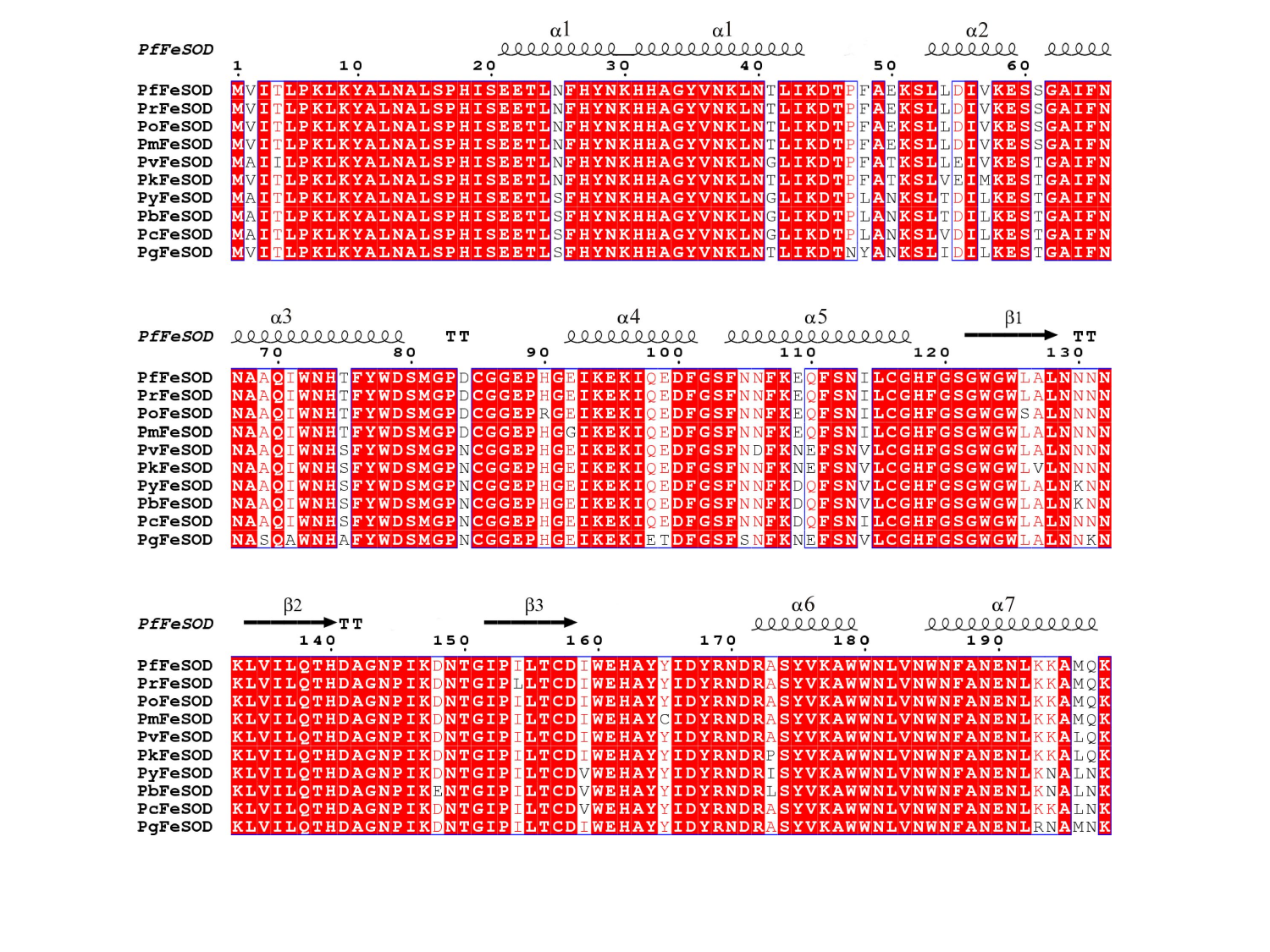

Supplement: Additional File 2 — Sequence alignment of Plasmodia FeSOD sequences. FeSOD is one the most highly conserved proteins across different Plasmodium species [40]. Identical residues are highlighted with a red background and similar residues are boxed. PfFeSOD secondary structure elements are superposed. Codes: Pf, P. falciparum; Pr, P. reichenowi; Po, P. ovalae; Pm, P. malariae; Pv, P. vivax; Pk, P. knowlesi; Py, P. yeolii; Pb, P. berghei; Pc, P. chabaudi; Pg, P. gallinaceum. The figure was made using ESPript [38]. [file 1472-6807-6-20-S2.ppt]

## Slide 1
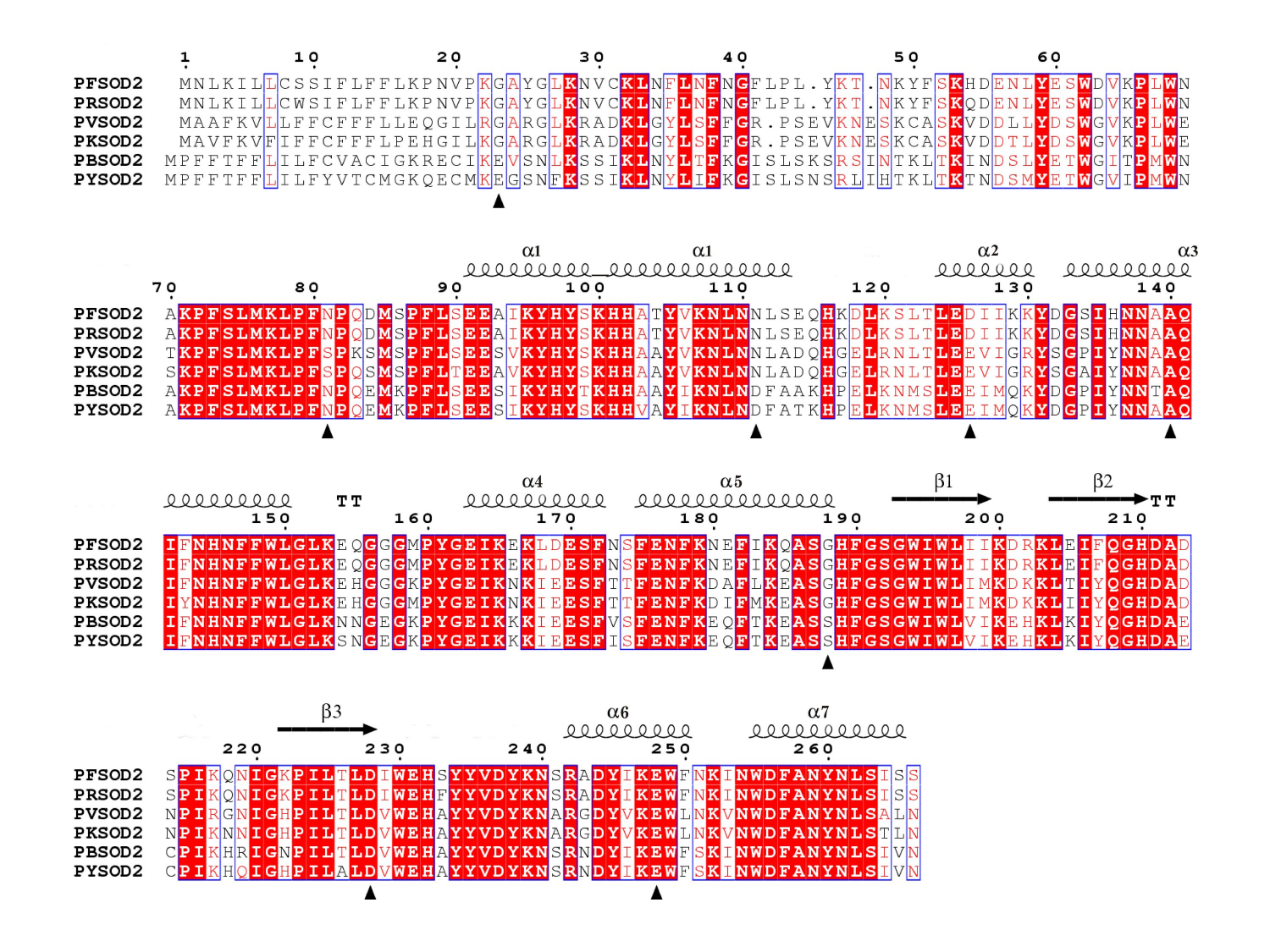

Supplement: Additional File 4 — Sequence alignment of predicted PfFeSOD2 proteins from Plasmodium species. Identical residues are highlighted with a red background and similar residues are boxed. PfFeSOD2 predicted secondary structure elements are superposed and exon boundaries are marked with a triangle. Note that the first two exons coincide with the putative signal sequence and transit peptide. Sequence data were gleaned from PlasmoDB [14]. Although not shown, the incomplete sequence data from P. chabaudi (missing region around exons 4 & 5) and P. gallinaceum (missing region encompassing 6 & 7) also show the same conserved pattern of putative intron boundaries. Codes: Pf, P. falciparum; Pr, P. reichenowi; Pv, P. vivax; Pk, P. knowlesi; Pb, P. berghei; Py, P. yeolii. [file 1472-6807-6-20-S4.ppt]
