# Supplementary material for: The crystal structure of superoxide dismutase from Plasmodium falciparum
Source: BMC Struct Biol. 2006 Oct 4;6:20. doi: 10.1186/1472-6807-6-20 (PMC1618392; doi:10.1186/1472-6807-6-20)
Supplement: Additional File 3 — Gene model of PfFeSOD2. A region of DNA sequence from P. falciparum chromosome 6 is shown, with predicted introns highlighted in yellow, and the protein product of the exon sequences in red. [file 1472-6807-6-20-S3.pdf]

P. falciparum chromosome 6 (complement 1000820-996029)

```
   1 TAATTTGTTTGAATATTATAATATATTATTTAATATATATATATATATAT
  51 ATATATATATATAATTGTATAATTAAAAAAAATATATATAATTAATGAAA
 101 TCATAACTTAAATGATTAAAAATGATTACTAATGTAATTATATATGTGAT
 151 TTAAATTTTTTTTTTTTTTTTTTTTATTTTACATACTATATATTTATAAATA
 201 TATTATATATATATAATATATATATTTATATATATATATTTATATAATAT
 251 ACATTTATGTGCCTTTGTAATTTTTTTTGTCAACGTATTCTTTTGATTGA
 301 AAAAAAGGAAAAAAAAAAAAAAATAAATAAAAAAATAAAAAAAAAAAAAA
 351 AAAAAAAAAAGAAAAAAGAATATAACATTATATTTGATGTTTTAAACCTT
 401 TTTCTTTTATATATATATATATATATATATGCATAGTATTTTATCTTTTC
 451 TTATTATGAGTATATAATATTTTTAAATAAAAACTACATATCAGCAGAAC
 501 TGTGTACCACTAAGAATTAGAAAACATGTATGAATATTATGACAATAATA
 551 AAAAAATTAGAAAACACCCACAAAAAGAAAAAAAAAAAAAAAAAAAAAAA
 601 ACTAAGAAATGTTATATTATATTATATTATATATAATAATATAATTTATT
 651 CATATTTATTAAGAAAGGCAATTATTTAATACTGTGATAATATCAACATT
 701 CATTTGTCATATGAATTCTAAAAAAGGATACTAAATAAATATATGTATAT
 751 ATTATTATGTATGTGTATTTTTTTTTTTATTTTTTATATCATTTTTTTTTT
 801 TTTTTTTTTTTTTTTTGGTATCTTCATATATTTTAAAAATATATATTTTA
 851 TACCATTTATAAATTTGAAAGAGAAAAATAAAATATTTTTATATTAAATA
 901 AAAAAAAAATATATATTATATATATATATATATATATATATATATTTTTA
 951 CAATATTATTTTTGTTCATGTTATATAAATTATATCTTTCGAATTACAAA
1001 ATGAATTTGAAGATTTTACTTTGTTCGTCAATTTTTTTGTTTTTTCTAAA
     M  N  L  K  I  L  L  C  S  S  I  F  L  F  F  L  K
      *  I  *  R  F  Y  F  V  R  Q  F  F  C  F  F  *  N
       E  F  E  D  F  T  L  F  V  N  F  F  V  F  S  K
1051 ACCAAATGTTCCCAAAGGTTCAAGAGCAATATATTTATTTTAACATTTTA
      P  N  V  P  K  G  S  R  A  I  Y  L  F  *  H  F  K
       Q  M  F  P  K  V  Q  E  Q  Y  I  Y  F  N  I  L
     T  K  C  S  Q  R  F  K  S  N  I  F  I  L  T  F  *
1101 AGATGAAAAAAAAATATACCACATTATAAATATATATATATATATATATA
       M  K  K  K  Y  T  T  L  *  I  Y  I  Y  I  Y  I
     R  *  K  K  N  I  P  H  Y  K  Y  I  Y  I  Y  I  Y
      D  E  K  K  I  Y  H  I  I  N  I  Y  I  Y  I  Y  I
1151 TATTTATATATAATACATTTTTGTAATATTTAATAGGTGCTTATGGGTTG
     Y  L  Y  I  I  H  F  C  N  I  *  *  V  L  M  G  *
      I  Y  I  *  Y  I  F  V  I  F  N  R  C  L  W  V  E
       F  I  Y  N  T  F  L  *  Y  L  I  G  A  Y  G  L
1201 AAAAATGTATGCAAGTTAAATTTTTTAAACTTTAATGGTTTTCTTCCTCT
      K  M  Y  A  S  *  I  F  *  T  L  M  V  F  F  L  Y
       K  C  M  Q  V  K  F  F  K  L  *  W  F  S  S  S
     K  N  V  C  K  L  N  F  L  N  F  N  G  F  L  P  L
1251 ATATAAAACTAATAAATATTTTTCTAAACATGATGAAAATTTATATGAAA
       I  K  L  I  N  I  F  L  N  M  M  K  I  Y  M  K
     I  *  N  *  *  I  F  F  *  T  *  *  K  F  I  *  K
      Y  K  T  N  K  Y  F  S  K  H  D  E  N  L  Y  E  S
1301 GTTGGGATGTTAAGCCATTATGGAATGCTAAACCATTTTCTTTAATGAAA
     V  G  M  L  S  H  Y  G  M  L  N  H  F  L  *  *  N
      L  G  C  *  A  I  M  E  C  *  T  I  F  F  N  E  I
       W  D  V  K  P  L  W  N  A  K  P  F  S  L  M  K
1351 TTGCCCTTCGTAAACATAAAAAAAAAAAGATTAATACAATATATAATCTTT
      C  P  S  *  T  *  K  K  K  I  N  T  I  Y  N  L  Y
       A  L  R  K  H  K  K  K  R  L  I  Q  Y  I  I  F
     L  P  F  V  N  I  K  K  K  D  *  Y  N  I  *  S  L
1401 ATTTTTAAAGTTATTATATATGTCTACATAAAAATAATTATATATATATA
       F  *  S  Y  Y  I  C  L  H  K  N  N  Y  I  Y  I
     I  F  K  V  I  I  Y  V  Y  I  K  I  I  I  Y  I  *
      F  L  K  L  L  Y  M  S  T  *  K  *  L  Y  I  Y  K
1451 AATATATGTATGTATGTATGTATTTTTTTTTTTATATTATTTATTATTTT
     N  I  C  M  Y  V  C  I  F  F  F  I  L  F  I  I  F
```

```
      I  Y  V  C  M  Y  V  F  F  F  L  Y  Y  L  L  F  F
       Y  M  Y  V  C  M  Y  F  F  F  Y  I  I  Y  Y  F
1501 TTTTTTTTTCTATAGAACCCTCAAGATATGTCACCTTTCTTGAGTGAGGA
      F  F  F  Y  R  T  L  K  I  C  H  L  S  *  V  R  K
       F  F  S  I  E  P  S  R  Y  V  T  F  L  E  *  G
     F  F  F  L  *  N  P  Q  D  M  S  P  F  L  S  E  E
1551 AGCTATAAAATATCATTATAGCAAACATCATGCTACATATGTGAAGAACT
       L  *  N  I  I  I  A  N  I  M  L  H  M  *  R  T
     S  Y  K  I  S  L  *  Q  T  S  C  Y  I  C  E  E  L
      A  I  K  Y  H  Y  S  K  H  H  A  T  Y  V  K  N  L
1601 TAAATAGTAAAAAGGAAAAAATTATATGTATATATATATATATTTATATA
     *  I  V  K  R  K  K  L  Y  V  Y  I  Y  I  F  I  Y
      K  *  *  K  G  K  N  Y  M  Y  I  Y  I  Y  L  Y  I
       N  S  K  K  E  K  I  I  C  I  Y  I  Y  I  Y  I
1651 TTTATTTATTTATTATGCTAATGTTTTATGTAATTATATGAAATTATTCC
      L  F  I  Y  Y  A  N  V  L  C  N  Y  M  K  L  F  L
       Y  L  F  I  M  L  M  F  Y  V  I  I  *  N  Y  S
     F  I  Y  L  L  C  *  C  F  M  *  L  Y  E  I  I  P
1701 TATAAACATTTTTATTTTTATTTTTATTTTTATTTTTTTTTGAAGATTTA
       *  T  F  L  F  L  F  L  F  L  F  F  F  E  N  L
     Y  K  H  F  Y  F  Y  F  Y  F  Y  F  F  L  K  I  Y
      I  N  I  F  I  F  I  F  I  F  I  F  F  *  R  F  I
1751 TCTGAGCAACATAAGGATTTAAAAAGTTTAACATTAGAAGGTAAGTGTGT
     S  E  Q  H  K  D  L  K  S  L  T  L  E  G  K  C  V
      L  S  N  I  R  I  *  K  V  *  H  *  K  V  S  V  L
       *  A  T  *  G  F  K  K  F  N  I  R  R  *  V  C
1801 TAATATTTATATTTTATATACTTTGAGTTCTATCTTTTCTTTTATGTTTA
      N  I  Y  I  L  Y  T  L  S  S  I  F  S  F  M  F  I
       I  F  I  F  Y  I  L  *  V  L  S  F  L  L  C  L
     *  Y  L  Y  F  I  Y  F  E  F  Y  L  F  F  Y  V  Y
1851 TATAATATTAACACGCTTTTTTTATTCAAAAAAATTCAGATATAATAAAA
       *  Y  *  H  A  F  F  I  Q  K  N  S  D  I  I  K
     Y  N  I  N  T  L  F  L  F  K  K  I  Q  I  *  *  K
      I  I  L  T  R  F  F  Y  S  K  K  F  R  Y  N  K  K
1901 AAGTATGATGGATCTATACACAATAATGCAGGTAATAAATATTACATATT
     K  Y  D  G  S  I  H  N  N  A  G  N  K  Y  Y  I  F
      S  M  M  D  L  Y  T  I  M  Q  V  I  N  I  T  Y  F
       V  *  W  I  Y  T  Q  *  C  R  *  *  I  L  H  I
1951 TCGTAAAACATATTTCATATATACACAAAATTTTATGCATCAAAAGTATG
      R  K  T  Y  F  I  Y  T  Q  N  F  M  H  Q  K  Y  E
       V  K  H  I  S  Y  I  H  K  I  L  C  I  K  S  M
     S  *  N  I  F  H  I  Y  T  K  F  Y  A  S  K  V  *
2001 AAAAGGATGTACATTGAATTATATACATATATATATAAACATTTTAATGT
       K  D  V  H  *  I  I  Y  I  Y  I  *  T  F  *  C
     K  R  M  Y  I  E  L  Y  T  Y  I  Y  K  H  F  N  V
      K  G  C  T  L  N  Y  I  H  I  Y  I  N  I  L  M  F
2051 TTTTTTTTTTTTTTTTATTTATATAGAATATAAAATATTTATCATATATAT
     F  F  F  F  F  I  Y  I  E  Y  K  I  F  I  I  Y  M
      F  F  F  F  L  F  I  *  N  I  K  Y  L  S  Y  I  C
       F  F  F  F  Y  L  Y  R  I  *  N  I  Y  H  I  Y
2101 GTACAATTATATTTATGTATATCTATATTTTATAAATGTGAAAAATATTG
      Y  N  Y  I  Y  V  Y  L  Y  F  I  N  V  K  N  I  D
       T  I  I  F  M  Y  I  Y  I  L  *  M  *  K  I  L
     V  Q  L  Y  L  C  I  S  I  F  Y  K  C  E  K  Y  *
2151 ACATTTTATAATTCTTTATTATTTTTCATTTTTTAATAATTCCTTTTTTT
       I  L  *  F  F  I  I  F  H  F  L  I  I  P  F  F
     T  F  Y  N  S  L  L  F  F  I  F  *  *  F  L  F  F
      H  F  I  I  L  Y  Y  F  S  F  F  N  N  S  F  F  F
2201 TTTTTTTTTTTTTTTTTTTTTGGTTAGCTCAAATTTTTAACCACAATTTCTT
     F  F  F  F  F  F  W  L  A  Q  I  F  N  H  N  F  F
      F  F  F  F  F  F  G  *  L  K  F  L  T  T  I  S  F
```

```
       F  F  F  F  F  L  V  S  S  N  F  *  P  Q  F  L
2251 TTGGCTGGGATTAAAAGAACAAGGTGGAGGAATGCCATATGGCGAAATAA
      W  L  G  L  K  E  Q  G  G  G  M  P  Y  G  E  I  K
       G  W  D  *  K  N  K  V  E  E  C  H  M  A  K  *
     L  A  G  I  K  R  T  R  W  R  N  A  I  W  R  N  K
2301 AAGAAAAACTTGATGAATCCTTCAATTCATTTGAAAATTTTAAAAACGAA
       E  K  L  D  E  S  F  N  S  F  E  N  F  K  N  E
     K  K  N  L  M  N  P  S  I  H  L  K  I  L  K  T  N
      R  K  T  *  *  I  L  Q  F  I  *  K  F  *  K  R  I
2351 TTCATAAAGCAAGCATCAGGTATAAATAAATAAAAGAATAAATATATACA
     F  I  K  Q  A  S  G  I  N  K  *  K  N  K  Y  I  H
      S  *  S  K  H  Q  V  *  I  N  K  R  I  N  I  Y  T
       H  K  A  S  I  R  Y  K  *  I  K  E  *  I  Y  T
2401 CATATATATATACATATATATATATTATTATCATATAATTTTAAAACAAA
      I  Y  I  Y  I  Y  I  Y  Y  Y  H  I  I  L  K  Q  N
       Y  I  Y  T  Y  I  Y  I  I  I  I  *  F  *  N  K
     H  I  Y  I  H  I  Y  I  L  L  S  Y  N  F  K  T  K
2451 ATTAACAGGTCATTTTGGAAGTGGGTGGATATGGTTAATAATAAAAGACA
       *  Q  V  I  L  E  V  G  G  Y  G  *  *  *  K  T
     I  N  R  S  F  W  K  W  V  D  M  V  N  N  K  R  Q
      L  T  G  H  F  G  S  G  W  I  W  L  I  I  K  D  R
2501 GAAAATTAGAGATTTTTCAAGGGCATGATGCTGATAGTCCTATTAAACAA
     E  N  *  R  F  F  K  G  M  M  L  I  V  L  L  N  K
      K  I  R  D  F  S  R  A  *  C  *  *  S  Y  *  T  K
       K  L  E  I  F  Q  G  H  D  A  D  S  P  I  K  Q
2551 AATATTGGAAAACCAATATTAACATTAGGTAAATAGGAACAGAAAATAAC
      I  L  E  N  Q  Y  *  H  *  V  N  R  N  R  K  *  Q
       Y  W  K  T  N  I  N  I  R  *  I  G  T  E  N  N
     N  I  G  K  P  I  L  T  L  G  K  *  E  Q  K  I  T
2601 AACAAAAAAAAAAAAAATATATATATATATATATATACATGTATATATTA
       Q  K  K  K  K  Y  I  Y  I  Y  I  Y  M  Y  I  L
     N  K  K  K  K  N  I  Y  I  Y  I  Y  T  C  I  Y  Y
      T  K  K  K  K  I  Y  I  Y  I  Y  I  H  V  Y  I  I
2651 TTTGCCACATTTTTATTATTTCATTTTTTAAAGATATATGGGAACATTCT
     F  A  T  F  L  L  F  H  F  L  K  I  Y  G  N  I  L
      L  P  H  F  Y  Y  F  I  F  *  R  Y  M  G  T  F  L
       C  H  I  F  I  I  S  F  F  K  D  I  W  E  H  S
1701 TATTATGTGGATTATAAAAACTCGAGAGCTGATTATATAAAAGGTTTGTG
      I  M  W  I  I  K  T  R  E  L  I  I  *  K  V  C  V
       L  C  G  L  *  K  L  E  S  *  L  Y  K  R  F  V
     Y  Y  V  D  Y  K  N  S  R  A  D  Y  I  K  G  L  C
1751 TAAAAATAGTTTTATAATGAATATATAAAATGCATTTCACGAATAATCAT
       K  I  V  L  *  *  I  Y  K  M  H  F  T  N  N  H
     *  K  *  F  Y  N  E  Y  I  K  C  I  S  R  I  I  I
      K  N  S  F  I  M  N  I  *  N  A  F  H  E  *  S  *
1801 AATATAATCATTCATAAAATAATTATATTATATATATATATATATATATA
     N  I  I  I  H  K  I  I  I  L  Y  I  Y  I  Y  I  Y
      I  *  S  F  I  K  *  L  Y  Y  I  Y  I  Y  I  Y  I
       Y  N  H  S  *  N  N  Y  I  I  Y  I  Y  I  Y  I
1851 TAATATATATATATGTTTTTTTTTTTTTTTTTAGAATGGTTTAATAAAAT
      N  I  Y  I  C  F  F  F  F  F  *  N  G  L  I  K  *
       I  Y  I  Y  V  F  F  F  F  F  R  M  V  *  *  N
     *  Y  I  Y  M  F  F  F  F  F  L  E  W  F  N  K  I
1901 AAATTGGGATTTTGCCAATTATAATTTGTCCATTTCAAGTTAAAATATAC
       I  G  I  L  P  I  I  I  C  P  F  Q  V  K  I  Y
     K  L  G  F  C  Q  L  *  F  V  H  F  K  L  K  Y  T
      N  W  D  F  A  N  Y  N  L  S  I  S  S  *  N  I  Q
1951 AAAAAATTTTTCATTTTTTTCTTTTCTTTTTAATGTTATCTTTTGAATAA
2001 AATTCAATATTTTATATCATTTTCTATTTTTTTTTTTTTTTTTTTTTTTG
2051 AGTATGGTTAAATTTTTTAAAATAACAAATTTGTATGAATATCCTTTTTT
2101 TTTTTTTTTTTTTTTTTTTTTTTTTCCTTTTTTGAATTATTCTTATTAAT
```
